# P-646. Culture Positive Pneumococcal Pneumonia Requiring Hospital Admission from 2017-2022 at Eight US Children’s Hospitals

**DOI:** 10.1093/ofid/ofae631.843

**Published:** 2025-01-29

**Authors:** Eric Engstrom, Kristina G Hulten, William J Barson, Philana L Lin, Steven Dahl, Kacy A Ramirez, John S Bradley, Pia Pannaraj, Tina Q Tan, Jennifer Dien Bard, Sheldon L Kaplan

**Affiliations:** Baylor College of Medicine/Texas Children's Hospital, Houston, TX; Baylor College of Medicine, Houston, Texas; Ohio State University College of Medicine and Public Health and Nationwide Children's Hospital, Columbus, Ohio; UPMC Children's Hospital of PIttsburgh, Pittsburgh, Pennsylvania; University of Arkansas for Medical Sciences, Little Rock, Arkansas; Wake Forest School of Medicine, Oak Ridge, North Carolina; University of San Diego School of Medicine, Rady Children's Hospital, San Deigo, CA; University of California San Diego; Feinberg School of Medicine, Northwestern University, Chicago, Illinois; Children's Hospital Los Angeles; University of Southern California, Los Angeles, CA; Baylor College of Medicine, Houston, Texas

## Abstract

**Background:**

The incidence of pneumococcal pneumonia (PP) has decreased significantly since the introduction of pneumococcal conjugate vaccines (PCV7, PCV13), however, pneumococci still remain the most common cause of bacterial pneumonia in children. We evaluated the current clinical presentation, isolate serotypes, and isolate antibiotic susceptibility of PP cases from 2017-2022.

**Figure:**
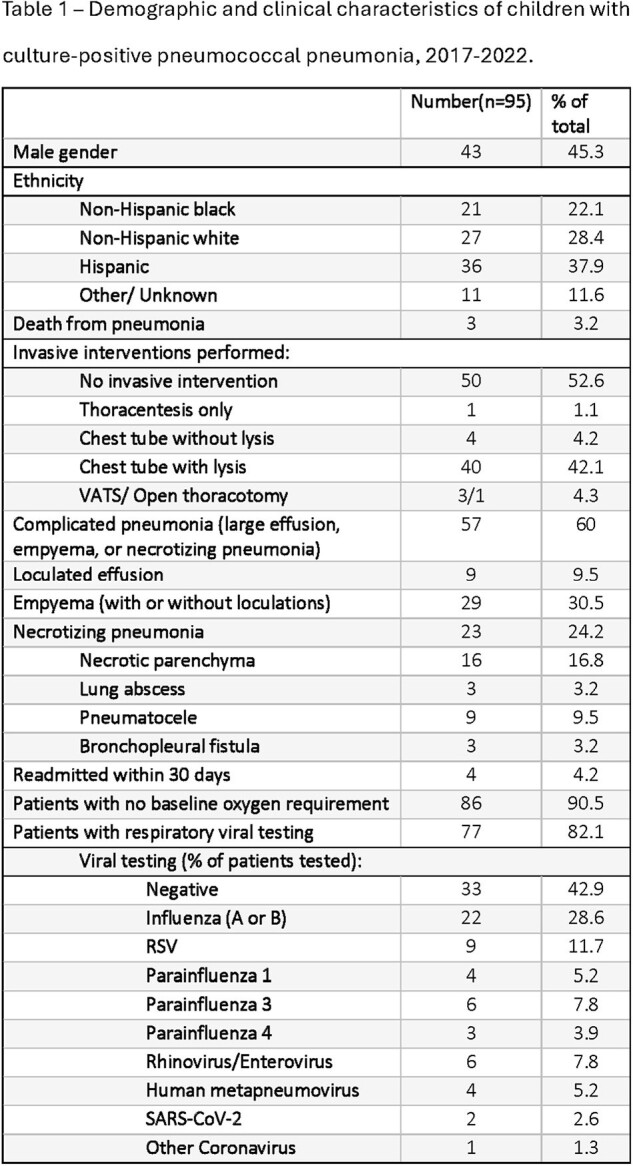

**Methods:**

We collected pneumococcal isolates and limited clinical data prospectively from patients with invasive pneumococcal disease ages ≤18 years at 8 children’s hospitals in the United States. Serotyping was performed at a central research laboratory. Patients with PP were identified and further clinical and laboratory data for each episode of pneumonia was collected retrospectively with a standardized case report form.

**Figure:**
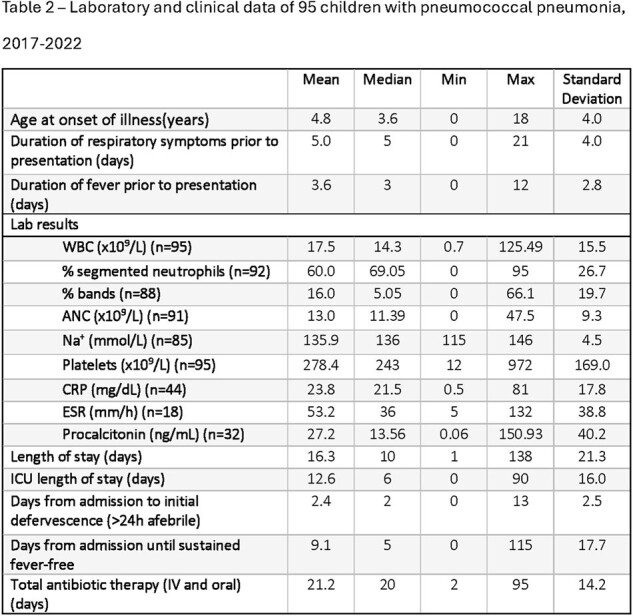

**Results:**

We collected 159 pneumococcal isolates and clinical data from patients with PP over the study period; extended clinical data was available for 95 patients (Figure 1). 9/159 (5.8%) isolates were non-susceptible to ceftriaxone (MIC > 1.0 µg/mL; 7/9 were 19A), and 10/159 (6.5%) were non-susceptible to penicillin (MIC > 2.0 µg/mL; 10/10 were 19A). 20/159 (12.6%) patients were completely unimmunized, while 108/159 (67.9%) were fully immunized with PCV7 or PCV13. Serotypes unique to PCV20 caused 34 episodes (21.4% of all isolates) (Figure 2). The risk ratio of a patient having PCV13 vaccine serotypes vs non-vaccine serotypes was not different between fully immunized and unimmunized (p=0.23), fully immunized and under-immunized by age (p=0.59), or any vaccination and unimmunized (p=0.11) patients. Of patients with extended clinical data, 40% had uncomplicated pneumonia, 31% had empyema (14/29-serotype 3, 6/29-19A, 1/29-19F), and 24% had necrotizing pneumonia (15/23-serotype 3, 4/23-19F, 0/23-19A). 78/95 (82.1%) patients had respiratory viral testing, of which 57.1% were positive for at least one virus (Table 1 and 2).

**Figure:**
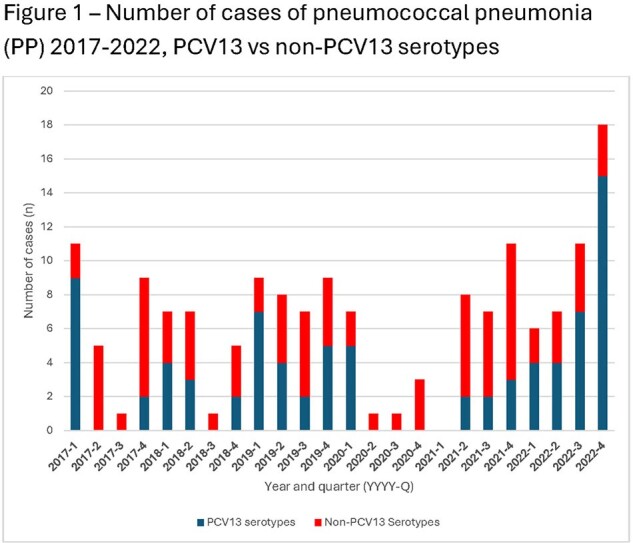

**Conclusion:**

PP continues to be an important cause of morbidity in the United States. Antibiotic resistance is a major concern for serotype 19A, but overall antibiotic resistance in pneumococcal pneumonia is low. The introduction of PCV20 in 2023 for routine administration to infants may modestly reduce incidence of PP.

**Figure:**
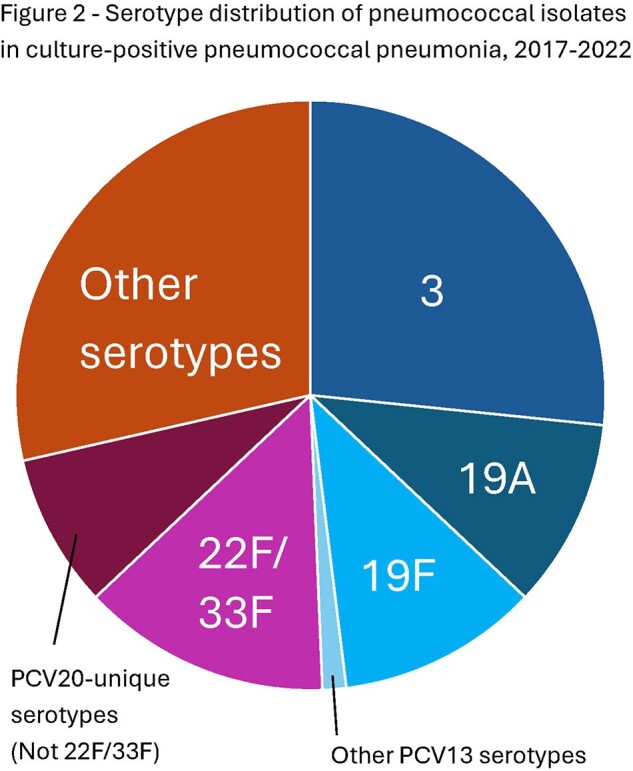

**Disclosures:**

**Kristina G. Hulten, PhD**, Pfizer: Advisor/Consultant|Pfizer: Grant/Research Support **William J. Barson, MD**, Pfizer: Grant/Research Support **Pia Pannaraj, MD, MPH**, AstraZeneca: Grant/Research Support|Pfizer: Grant/Research Support **Tina Q. Tan, MD**, Astra Zeneca: Grant/Research Support|GSK: Grant/Research Support|Iliad: Advisor/Consultant|Merck: Advisor/Consultant|Moderna: Advisor/Consultant|Novavax: Advisor/Consultant|Pfizer: Advisor/Consultant|Sanofi Pasteur: Advisor/Consultant **Sheldon L. Kaplan, MD**, Pfizer: Grant/Research Support

